# Transmission of Grapevine Ampelo- and Vitiviruses by the Bohemian Mealybug *Heliococcus bohemicus* Šulc (*Hemiptera: Pseudococcidae*)

**DOI:** 10.3390/v14071430

**Published:** 2022-06-29

**Authors:** Gérard Hommay, Monique Beuve, Etienne Herrbach

**Affiliations:** UMR 1131 Santé de la Vigne et Qualité du Vin, INRAE, Université de Strasbourg, F-68000 Colmar, France; gerard.hommay@inrae.fr (G.H.); monique.beuve@inrae.fr (M.B.)

**Keywords:** mealybug, *Vitis vinifera*, leafroll, rugose wood, GLRaV, GVA, virus transmission

## Abstract

Grapevine-infecting ampelo- and vitiviruses are transmitted by several scale insect species, including the Bohemian mealybug, *Heliococcus bohemicus* Šulc. Virus infectivity experiments were performed with this species to study the transmission ability of natural populations living in infected vineyards in Alsace, France. Mealybugs were sampled on vines infected by grapevine leafroll-associated viruses (GLRaV-1, -2, and -3) and by grapevine virus A (GVA), either alone or in combinations. Out of six natural populations tested, only one, located at Bennwihr, was able to transmit GLRaV-1 and -3 to healthy vines, though with low transmission rates (1.6 and 11.8%, respectively). Mealybugs from Bennwihr were also able to transmit GLRaV-3 from grapevines of another location where *H. bohemicus* was not a vector. Conversely, mealybugs from two other locations did not transmit any virus acquired from infected grapevines at Bennwihr. These results suggest differences in vector ability between *H. bohemicus* populations. Moreover, laboratory experiments were developed to estimate the minimal acquisition and inoculation access periods (AAP and IAP, respectively) for virus transmission of GLRaV-1 and -3, and GVA. First instar nymphs transmitted GLRaV-1 after 6 h AAP, GLRaV-3 and GVA together after 1 h AAP, and the three viruses after only 1 h IAP, supporting a semi-persistent mode of transmission. Second instar nymphs fed on multi-infected grapevine for 72 h then starved or fed on potatoes tested positive by RT-PCR for GLRaV-1 and -3 after up to 35 and 40 days, respectively, contrasting with the short retention times generally observed for mealybugs. These findings provide new knowledge of the vector ability of *H. bohemicus*.

## 1. Introduction

Grapevine leafroll disease (GLD) is one of the most important grapevine viral diseases and occurs in all major grapevine-growing areas. At least four serologically distinct *Closteroviridae* species, designated as *Grapevine leafroll-associated virus* (GLRaV) -1, -2, -3, and -4, are associated with GLD, among which three (GLRaV-1, -3, and -4, genus *Ampelovirus*) are naturally transmitted by mealybugs (*Hemiptera: Pseudococcidae*) and soft scales (*Hemiptera: Coccidae*) [1,2]. Moreover, three further virus species, *Grapevine virus A* (GVA), GVB, and GVE (genus *Vitivirus*, family *Betaflexiviridae*) associated with the “rugose wood” (RW) complex, are also transmissible by some of these vector species [2]. To date, in France, only GLRaV-1, -2, and -3, and GVA have been identified in naturally infected vineyards [3]. GLD can cause delay in fruit maturation and severe reductions in quality and yield [4,5]. RW may reduce vigour and shorten longevity of vines, but the question of how much vitiviruses affect production is still to be fully answered [6]. Grapevine ampelo- and vitiviruses are restricted to phloem tissues. Most studies dealing with acquisition, retention, and inoculation of these viruses show that they are transmitted in a semi-persistent non-circulative manner [7,8,9,10]. Indeed, there is a short latency (a few hours), and neither transstadial nor transovarian passage (reviewed by [1]). Several mealybug species can transmit a given ampelovirus, whereas one mealybug species is capable of transmitting different ampeloviruses. Therefore, Garau et al. [11], Tsai et al. [12], and Le Maguet et al. [13] have suggested a lack of a strict virus–vector specificity of transmission between different mealybug species and virus species.

The Bohemian mealybug, *Heliococcus bohemicus* Šulc, is a highly polyphagous species, widely distributed in the Palaearctic region from Western Europe to China [14]. It occurs in the vineyards of France, Germany, Hungary, and Northern Italy [15,16,17,18,19,20,21,22]. *H. bohemicus* has been identified as a vector of GLRaV-1 and -3, and GVA [21,23,24]. In the vineyards of north-eastern France and south-western Germany, three species of soft scales, *Parthenolecanium corni* (Bouché), *Parthenolecanium persicae* (Fabricius), and *Pulvinaria vitis* (L.), and two of mealybugs, *H. bohemicus* and *Phenacoccus aceris* (Signoret), can be found [19,21,25]. After *Pa. corni*, *H. bohemicus* is the most common species. However, it seems more localised and is usually not considered a pest, though populations can sometimes reach high numbers, partly due to reductions in insecticide treatments. The backs of nymphs and adult females have thin and long glassy wax filaments, which differentiate them from *Ph. aceris*. *H. bohemicus* is bisexual and develops only one generation per year in our region (monovoltinism) compared with two in Italy [17,20]. Second instar nymphs (L2) and adults overwinter under the stock bark. In April, they migrate to feed on buds, while the L2 remaining under the bark moult into a ‘pupa’ inside a structure called a ‘puparium’ from which emerge winged males [26]. After mating, females, which are ovoviviparous, migrate back under the bark to give birth to first instar nymphs (L1), which remain agglutinated for a few days under and near the mother’s body. Then, the L1 move up the trunk to colonise basal leaves around mid-June. During summer they moult into L2, which scatter throughout the grapevine foliage. In October, before leaf fall, the L2 move down to overwinter under the bark [26]. Virus transmission by natural populations of *H. bohemicus* in north-eastern France is little-documented [21,27]. In other virus-transmitting scale insect species, L1 were reported to be more efficient vectors than L2, L3 (if this stage exists), and adults [1]. To improve our knowledge of the ability of this species to transmit GLD viruses and GVA, and to characterise the features of this transmission, we performed experiments with populations from several vineyards, using (1) infectivity assays, where natural populations were sampled from infected vineyards and transferred onto healthy recipient vines in the laboratory, and (2) controlled transmission experiments in the laboratory.

## 2. Materials and Methods

**Figure 1:**
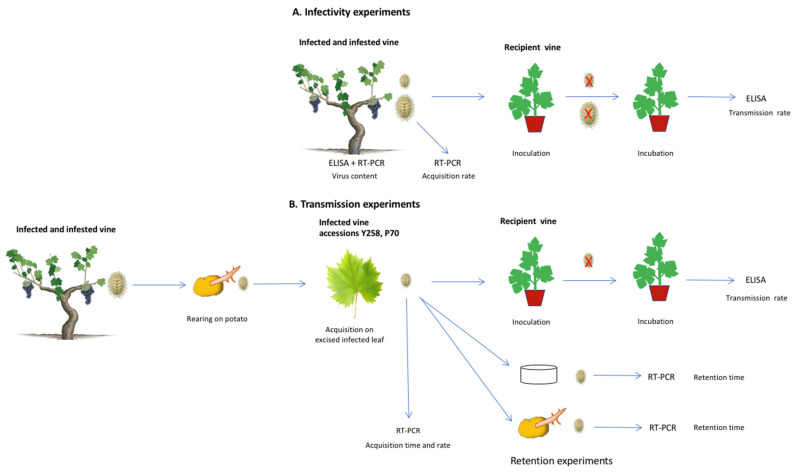
Graphical description of the different experiments performed. (**A**): Infectivity experiments; (**B**): Controlled transmission and retention experiments.

The various experimental approaches used in studying the vector biology of *H. bohemicus* are schematised in Figure 1.

### 2.1. Infectivity Experiments

Inoculation experiments (Figure 1A) were conducted to study the transmission ability of natural populations sampled from infected vineyard vines, at different larval stages.

- Origin of insects and viruses

The *H. bohemicus* populations originated from commercial vine plots at six locations in Alsace (north-eastern France): Bennwihr, Colmar, Kientzheim, Nothalten, Ribeauvillé, and Turckheim, where it was the only mealybug species present. Heavily infested grapevines were first tested by enzyme-linked immunosorbent assay (ELISA) for GLRaV-1, -2, and -3, and GVA (Table 1) before sampling mealybug-bearing leaves for infectivity experiments. Additional ELISA and reverse transcription polymerase chain reaction (RT-PCR) tests were performed for grapevines for which initial results were not clear-cut. Each grapevine tested was identified by its spatial location (row number, stock number). The distribution of viruses among the grapevines tested within each plot is given in Table 1. We attempted to obtain a representative number of the different virus combinations among source grapevines hosting *H. bohemicus* colonies. GLRaV-2 being rare, associations with this virus were few.

For a portion of the plants tested, a multiplex reverse transcription polymerase chain reaction (RT-PCR) was used to detect GLRaV-1, -2, and -3, and GVA in insect samples (1 to 74 L2) before infectivity experiments, using the following primers for GLRaV-1 (‘LR1-H70F1’ GTTGGTGAATTCTCCGTTCGT and ‘LR1-H70R1’ ACTTCGCTTGAACGAGTTATAC), GLRaV-2 (‘P19qtF’ ATGGAGTATTGTTTGAAGCAGGTAC and ‘P24qtR’ AGAATGTCTTCAGCTTCATAAGGAG), GLRaV-3 (‘LR3-POLF1’ ACGTAACGGGGCAGAATATAGT and ‘LR3-POLR1’ TATCAACACCAAGTGTCAAGAGTA), and GVA (‘GVA-CPF1’ GGCTACGACCGAAATATGTAC and ‘GVA-CPR1’ AGAAACGATGGGTCATCCATC), following the protocol developed by Beuve et al. [28]. Nymphs were stored in 1.5 mL Eppendorf tubes with 150 μL RLT buffer (RNeasy Plant Mini Kit™; Qiagen, Les Ulis, France) containing 1% β-mercaptoethanol, and were kept at −20 °C before total RNA extraction and detection of viruses by RT-PCR. PCR products were visualised under UV light on a 2% agarose gel stained with ethidium bromide. RNA extracts from GLRaV-1, -2, and -3, and GVA infected grapevines were used as virus positive controls, and RNAse-free water (Kit Qiagen™) was used as the negative control.

- Recipient grapevines

Virus-free grapevines were obtained from rooted cuttings of *Vitis vinifera* cv. Pinot noir, frequently planted in vineyards of Alsace, Champagne, and Burgundy (clones P114 and mainly P115), or from germinated pips of Pinot noir and Pinot blanc. Mother plants of the cuttings were tested with ELISA and RT-PCR for the absence of leafroll and rugose wood viruses (GLRaV-1, -2, and -3, and GVA). Plants were grown in individual pots under greenhouse conditions until the 6–12 leaf stage and then used in transmission experiments. They were sprayed bi-monthly with an insecticide (alternatively Confidor™ 0.35 mL/L, Kiros™ 1 mL/L, or Fuoro™ 3 mL/L) to ensure the absence of insects; this spraying was stopped at least one month before insect inoculation. A sub-sample of ten recipient plants was tested to determine their virus-free status by ELISA prior to transmission experiments.

- Transmission to healthy recipient grapevines

Since viruses are unevenly distributed on grapevines, several leaf pieces were cut from infected grapevines bearing first instars nymphs (L1) (2 to 100 individuals, mean ± sd = 34 ± 33) and attached with hairclips onto the leaves of virus-free recipient grapevines (3–4 leaf pieces per plant). Insects crawled off as the leaf fragments dried. As for the L2 nymphs, these were gently stimulated to move, then transferred with a fine paintbrush inside small cages (h = 8 mm, internal diameter = 13 mm). The cages were then attached with hairclips to leaves of the recipient plants. The number of recipient plants used according to the origin and cultivar of the virus combinations is given in Table 2. The numbers of nymphs used to settle on recipient plants varied greatly according to the numbers collected from the source grapevine (Bennwihr: 2 to 100 L2, mean ± sd = 32 ± 20; other locations: 1 to 60 L2, mean ± sd = 20 ± 16). Finally, batches of 2 to 70 overwintering L2 (mean ± sd = 33 ± 19) were collected between January to early April under the bark of infected vines from Bennwihr and transferred onto recipient vines at laboratory temperature. Each recipient plant was isolated from the others under a 0.1 mm mesh micro-perforated plastic bag (‘bread bags’, Sealed Air SAS, Épernon, France), firmly secured to the pot using a rubber band to prevent nymphs from migrating between plants. Transmission experiments were conducted at 20–23 °C, 16 h/8 h (L/D) under artificial light. After 5 to 7 days of the inoculation access period (IAP), source leaves or cages were withdrawn. Mealybugs on leaves were removed with a paintbrush, after which grapevines were immediately sprayed with mevinphos (4 mL/l Phosdrin W10™) to kill the remaining insects. After two days, the treated plants were checked for any surviving insects, then transferred into a glasshouse compartment dedicated to recipient plants only. Recipient grapevines with nymphs from uninfected grapevines grown under the same conditions were used as negative controls. In late November, the recipient grapevines were pruned back to two buds and kept under an unheated glasshouse for overwintering. In spring, they were transferred into a heated glasshouse. All recipient plants were periodically sprayed with insecticide and fungicide, and pruned to avoid overgrowth until the end of the study.

- Virus detection in grapevines

The infection of recipient vines was assessed by double-antibody sandwich enzyme-linked immunosorbent assay (DAS-ELISA). The grapevines were checked by ELISA 4–6 and 8–12 months after IAP, and up to 18–24 months for surviving plants that had previously remained negative. The plants inoculated in late September could not be tested before cold storage and were therefore tested first about 6–7 months after IAP. Regardless of the recipient grapevine, these were systematically tested for the presence of GLRaV-1 and -3 and GVA. GLRaV-2 was only tested for when the source grapevine had been infected with this virus. The accession Y258 (Armenian cv. Liali Bidona, also named Vardabuyr), multi-infected by GLRaV-1 and -3, and GVA, was used as a positive control for the three viruses. The accession Chardonnay V38, infected by GLRaV-2 and -3, was used as the positive control for GLRaV-2. Healthy P115 cuttings were used as negative controls. Tissue extracts were obtained from pooled fragments of three leaves. Leaf fragments were ground (1 g leaves for 5 mL buffer) inside extraction bags with a bullet blender (Homex 5™, Bioreba, Reinach, Switzerland). Polyclonal antibodies raised against GLRaV-1, -2, or -3, or GVA produced in the laboratory were used in a biotine-streptavidine procedure [29]. Absorbance values were recorded at 405 nm using a Multiskan™ microplate reader (Thermo Labsystems, Helsinki, Finland). Values above the mean of six healthy controls (six replicates per plate) plus three times their standard deviation were considered positive.

- Statistical analysis

Chi-square tests were used to compare the detection rates in L2 nymphs, according to virus species and life cycle. A *p*-value < 0.05 was considered as the threshold for significance. Statistical analyses were performed with R, version 2.10.

### 2.2. Controlled Transmission Experiments

Firstly, the rate of virus acquisition by L1 nymphs was investigated according to feeding time and to nymph numbers (Figure 1B). Secondly, the time of virus acquisition was refined with L2 nymphs. Based on the results obtained, optimal times for virus acquisition and inoculation were then investigated for these two stages. Lastly, the virus retention time of L2, either starved or fed on potatoes, was tested after 3 days AAP on infected grapevines (Figure 1B).

- Origin of insects and virus sources

The *H. bohemicus* culture originated from the Riesling plot at Colmar. L2 nymphs were collected in April from new leaf buds and raised on potato sprouts inside glass jars, closed with a 28 μm mesh tissue to enable airflow, and kept in the dark at 20–23 °C. When they became adults and gave birth to larval clusters, L1 nymphs were collected to determine their rate of virus acquisition according to feeding time and optimal AAP and IAP. L2 nymphs used to evaluate the virus acquisition rate according to feeding time were collected on the same plot and reared for at least one month on potato. Those used for optimal AAP/IAP and retention time were born on potato.

Virus source cuttings from accessions of our reference collection of grapevine viruses [30] were rooted in a greenhouse. Accession Y258 infected with GLRaV-1 and -3, and GVA was used as the virus source plant to determine minimal AAP and IAP by L1 and the rate of acquisition by L2 nymphs according to time. Accession P70 of Pinot noir infected with GLRaV-1 and GVA [31] was used as the virus source plant to determine minimal AAP and IAP by L2 nymphs. P70 was chosen because its viruses are well transmitted by *Pa. corni* [32] and *Phenacoccus aceris* (Signoret) [13]. The virus content of the source plants was checked by ELISA and RT-PCR prior to the experiments.

- Virus acquisition

Leaves of virus source grapevines were collected and placed individually into tight and round polystyrene crystal boxes. A small wet cotton piece was wrapped around the cut petiole of each leaf, then tightly swathed inside Parafilm^TM^ to retain the water supply during AAP. Mealybugs were collected with a fine paintbrush from sprouted potatoes and transferred onto the underside of source grapevines leaves for AAP.

*Rate of virus acquisition according to time of feeding*: AAP of L1 nymphs on Y258 leaves was tested for 24, 48, and 72 h. For each time, virus detection was conducted by RT-PCR on five samples of 5, 10, and 20 L1. For L2, AAP of 1, 3, 6, 24 h, and from 2 up to 7 days, was tested on leaves of Y258. Ten mealybugs per batch were chosen based on the results obtained with L1, and virus detection by RT-PCR was performed on ten batches of L2 for each AAP. The presence of viruses was checked in fragments of source leaves before AAP. A batch of ten mealybugs collected from potato rearing was used as a healthy control.

*Minimal time for virus acquisition and inoculation*: After 1, 6, or 24 h AAP on Y258 leaves for L1, and on P70 leaves for L2, nymphs were transferred into small cages, which were clipped onto the leaves of five virus-free recipient cuttings (P115) for 1, 6, or 24 h for IAP (30 nymphs per plant distributed on two leaves), allowing for each growth stage a total of 9 AAP × IAP pairs. Each recipient plant was enclosed in a bread bag, as previously described. Plants were placed at 20–23 °C, 16 h/8 h (L/D), under artificial light. After IAP, the recipient plants were immediately sprayed with an insecticide. Grapevine cuttings of the same age, without nymphs and kept in the same conditions under a glasshouse, were used as negative controls.

- Virus detection in recipient grapevines

Leaves of the recipient vines were tested by ELISA, as described above. First detection tests were conducted on leaves ca. 4 months after the transmission experiments. After a dormancy period under an unheated glasshouse, plants were tested again 10–12 months after IAP. Virus source grapevines and healthy cuttings were used as positive and negative controls, respectively.

- Comparison between populations

To compare inoculation capacities between *H. bohemicus* populations, L2 nymphs were collected in April from three plots located at Bennwihr, Turckheim, and Ribeauvillé, and reared separately on potato tubers. Inoculation experiments were conducted with these mealybugs (after a latent period of ≥two months on potato, a non-host for leafroll viruses, thus being considered to be non-viruliferous) and their progeny (virus-free, as leafroll viruses are not transovarially transmitted) in June–July and October. Mealybugs from Bennwihr were placed on leaves of infected grapevines from Nothalten (see Virus acquisition above), whereas those from Turckheim and Ribeauvillé were placed on leaves of infected grapevines from Bennwihr for an AAP of 7 days (Nothalten and Colmar populations were too low to allow testing). After AAP, mealybugs were transferred onto healthy P115 cuttings for an IAP of 7 days.

### 2.3. Virus Retention Time

L2 nymphs from potato rearing were allowed to feed on leaves of the multi-infected accession Y258 for a 72 h AAP. Then, they were collected, and one half thereof was starved inside an empty box, while the other half was transferred onto potato tubers. Virus content was then tested by RT-PCR, as described above, on five batches of ten mealybugs at times spanning daily from 7 to 15 days after transfer, then every ten days until 60 days for those on potato tubers and every five days until 35 days for those starving. Potatoes used as food supply were simultaneously tested by RT-PCR for virus content.

## 3. Results

### 3.1. Infectivity Experiments

#### 3.1.1. Virus Content in Vineyard-Sampled Mealybugs

For a set of source vines harbouring various virus combinations, virus content was tested by RT-PCR in the L2 nymphs, either active on leaves in spring or overwintering under stock bark. Positive detection was obtained in mealybug samples for the four viruses being tested, with smaller detection rates for GVA. Detection rates of GLRaV-1 (respectively 79 and 62%) were close to those of GLRaV-3 (respectively 68 and 63%) (Table 3). For the overwintering L2 nymphs, detection rates were significantly different between viruses (χ^2^ = 16.3, df = 3, *p* = 0.001) due to the lower rates for GLRaV-2 and GVA. The proportion of overwintering samples that tested positive for at least one virus was around 78% (Table 3) and varied little during winter (Table 4). The proportion of active samples that tested positive for at least one virus was not significantly higher, at 88% (Table 3; χ^2^ = 0.86, df = 1, *p* = 0.354). Winged adult male samples collected in April (n = 4 batches) tested negative, as expected, due to their inability to feed and the absence of transstadial virus transmission.

#### 3.1.2. Infectivity of Natural Populations

Vineyard-sampled mealybugs were transferred onto healthy recipient plants. After completing the IAP and the insecticide treatment, no living insects were found on recipient plants. Healthy control plants, with or without non-viruliferous mealybugs, were all negative in ELISA (Table 5 and Table 6). Transmission events were observed only with nymphs sampled from Bennwihr (Table 5 and Table 6), which transmitted GLRaV-1 and -3. Two GLRaV-3 transmission events were recorded in May with the L1; eleven others (one for GLRaV-1 and ten for GLRaV-3) were recorded with the L2 and IAPs spanning from August to September; and none were observed at all with the active L2 in October and early April, or in the overwintering L2 (Table 6). GLRaV-3 was transmitted from either a vine singly infected by this virus, or vines also co-infected by GLRaV-1 and GVA (Table 6). The lowest number of L2 per recipient plant for successful transmission was 22. First virus detection occurred around one year on average after IAP, with the earliest at 7.2 months. Even though most of the collected nymphs were positive for at least one virus (Table 3), transmission occurred only in a few cases (1/63 = 1.6% for GLRaV-1 and 12/102 = 11.8% for GLRaV-3). Whatever the origin and the cultivar of the source vine (Pinot noir, Riesling, or Sylvaner; Table 1), or of the recipient grapevines (P114, P115, Pinot noir, or Pinot blanc; Table 2), GLRaV-2 and GVA were not transmitted to healthy grapevines in our infectivity experiments (Table 5 and Table 6).

### 3.2. Controlled Transmission Experiments

#### 3.2.1. Comparison of *H. bohemicus* Populations as Vectors

With the aim of comparing the vector ability of various populations, adult females sampled at Bennwihr, Turckheim, and Ribeauvillé were reared on potato to allow them to give birth to nymphs. Thereafter, the L2 nymphs were placed for AAP on excised leaves of infected grapevines from Nothalten or Bennwihr (Table 7). Only Bennwihr nymphs transmitted GLRaV-3 from GLRaV-1 and/or -3 infected Nothalten vines to recipient cuttings. Those from Turckheim and Ribeauvillé placed on leaves of infected grapevines from Bennwihr did not transmit any virus.

#### 3.2.2. Rate of Virus Acquisition by L1 Nymphs According to Time

After various AAPs (24, 48, or 72 h), GLRaV-1 and GVA were detected by RT-PCR in batches of five L1 or more, whereas GLRaV-3 was detected in ten or 20 L1 per batch (Table 8). Globally, virus detection increased along with nymph numbers in the L1 batches and was better after 48 h or 72 h than after 24 h of AAP.

#### 3.2.3. Rate of Virus Acquisition by L2 Nymphs According to Time

**Figure 2:**
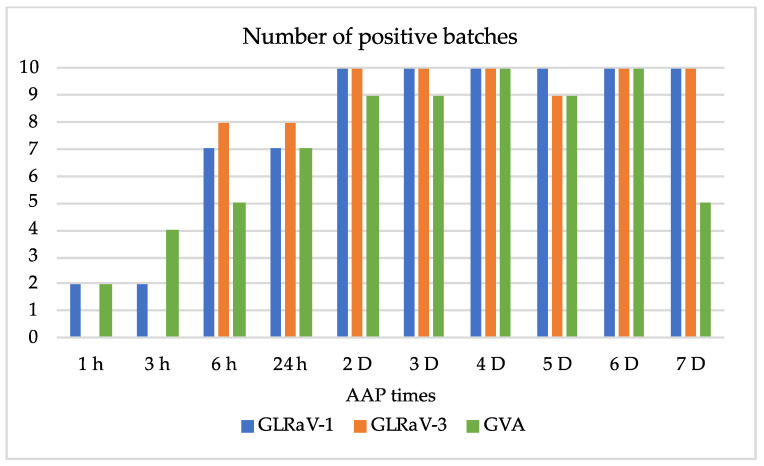
Virus detection rates in L2 nymphs of *Heliococcus bohemicus*, after 1 h to 7 days (D) AAP on the accession Y258, infected with GLRaV-1 and -3, and GVA.

GLRaV-1 and GVA were detected by RT-PCR in batches of L2 tested after 1 h and 3 h of AAP on leaves of the accession Y258, whereas GLRaV-3 was detected only after 6 h AAP (Figure 2). The maximal number of positive batches for GLRaV-1 and -3 was reached as early as two days AAP. Despite the number of ten batches tested, the evolution of positive numbers of batches was more irregular for GVA. In all experiments, source leaves were confirmed by ELISA to harbour the three viruses, and mealybugs from potato rearing were found to be negative following RT-PCR.

#### 3.2.4. Minimal Time for Virus Acquisition and Inoculation

Transmission experiments were performed by combining various AAP and IAP (Table 9). With the L1 feeding on Y258, 1 h AAP or IAP were sufficient for transmission of GLRaV-3 and GVA, whereas 6 h AAP was sufficient for transmission of GLRaV-1. The shortest time sequence of AAP/IAP for successful transmission of GLRaV-1 was 6 h/1 h. The shortest times of AAP/IAP for successful transmission of GLRaV-3 and GVA were 1 h/24 h or 24 h/1 h. No recipient grapevine was infected by the L2 that had fed on the P70 source vines whatever the combinations of AAP (1, 6, or 24 h) and IAP (1, 6, or 24 h).

### 3.3. Virus Retention in L2 Nymphs According to Diet

After a 3-day AAP on accession Y258, the L2 nymphs were tested by RT-PCR after various periods spent either starving or confined on potato. In batches of starved nymphs, GLRaV-1 and -3, and GVA RNAs were detected until 35 days after the start of starvation (Table 10). Those fed on potato tested positive for GLRaV-1 and -3, and GVA until 40 days after their transfer from the infected vines.

## 4. Discussion

### 4.1. Vector Efficiency of H. bohemicus Populations

Among six natural populations of the mealybug *H. bohemicus* distributed in infected vineyards of Alsace (France), only one, located at Bennwihr, was able to transmit GLRaV-1 and -3 to healthy grapevines in infectivity experiments. In addition, this population could transmit GLRV-3 when fed on infected vines from Nothalten, while the mealybug population from this location was non-vector. Conversely, non-vector mealybugs from populations from two other locations did not transmit viruses from the infected vines from Bennwihr. However, in nymphs living on mixed-infected grapevines from the six locations, all the viruses present could be detected, showing that nymphs fed well of phloem sap. Virus detection in mealybugs was thus not linked to their transmission ability. Moreover, the handling of nymphs might have affected their proneness to inoculate the virus. As expected, GLRaV-2 was detected in nymphs but was not transmitted. No species among soft scales is known to transmit GLRaV-2 [1,2]. GLRaV-2 is the sole grapevine virus, within the *Closteroviridae* family, assigned to the genus *Closterovirus*, of which the only known vectors are aphids [33]. The reported absence of GLRaV-1 or -3 transmission by our non-vector natural populations is unlikely to result from a low number of nymphs, since we used up to 100 L1 and 60 L2 per recipient vine. Moreover, Bertin et al. [24] obtained virus transmissions with only five *H. bohemicus* L1 or L2 nymphs per test plant.

A possible explanation for this differential vector ability between populations is that the receptors, which retain virions in the insect, may vary in their viral affinity. Geographic intraspecific genetic variations exist among mealybugs [34]. The low mobility of *H. bohemicus* could favour geographical differences between populations even though our sampling sites are located within a distance of ca. 40 km. In this context, it would be interesting to study whether different the transmission ability between *H. bohemicus* populations could result from genetic differences. To support this assumption, it would be interesting to precisely localise receptors where virions are retained before being released for inoculation, and compare their affinity capacities in vector and non-vector populations.

### 4.2. Possible Effect of Viral Variants

Virus species also present genetically and geographically distinct variants that can influence their transmission efficiency. The L1 from Colmar populations transmitted viruses from the accession Y258, while the L2 from the same population were unable to transmit viruses from the accession P70. In addition, mixed infections of viral variants within a single plant are common and differential transmission of variants may occur [35]. For example, Blaisdell et al. [36] observed that mixed infection from two singly infected source plants resulted in fewer mixed infections than expected by chance, which may be due to competition between virus variants. More generally, the variability of vector ability between populations of the same species has been reported, though it is difficult to determine the variability attributable to virus variants or/and to vector populations. Whereas Belli et al. [37] showed that *Pu. vitis* transmits GLRaV-3, Hommay et al. [38] recorded no transmission of this virus with *Pu. vitis* nymphs sampled on grapevines infected with various virus associations. In Europe, *Pa. corni* was shown to transmit GLRaV-1 but not GLRaV-3 [21,32,39], though GLRaV-3 transmission by this species was reported in Washington State, USA [40]. Variations of transmission rates within a given vector species are likely due to a combined effect of experimental conditions, vector and virus intraspecific variability, and differential susceptibility of grapevine varieties to either the virus or vector [41].

### 4.3. Seasonal Effects

The L1 and summer L2 nymphs from the Bennwihr population transmitted viruses, but no results were obtained with the L2 sampled during winter or in early spring before maturation into adults. The early spring L2 were probably disturbed when transferred for infectivity experiments, which could have reduced their feeding activity and anticipated moulting into adulthood as some gave birth to crawlers during experiments. Our transmission experiments with *H. bohemicus* specimens sampled in spring from the Colmar population showed that the L1 were able to transmit all three viruses, GLRaV-1 and -3, and GVA, after acquisition on the multi-infected accession Y258. However, the summer L2 from the same location were unable to transmit GLRaV-1 and GVA after AAP on the accession P70. These experiments were performed with the same infected and recipient plant accessions as in the transmission experiments with *Pa. corni* [32], *Ph. aceris* [13], and *Pu. vitis* [38], where both L1 and L2 of these two species successfully transmitted the viruses from P70. The reason why the *H. bohemicus* L2 were unable to do so remains unclear.

### 4.4. Transmission Efficiency

Sforza et al. [21] reported that groups of 30–50 individuals carrying GLRaV-1 and -3 were able to transmit at least one of these viruses to 14% of recipient plants. Bertin et al. [24] observed that, although the proportion of *H. bohemicus* nymphs that acquired at least one virus was high (88 to 100%), virus transmission occurred at low rates (26%). Zorloni et al. [23] showed that *H. bohemicus* nymphs transmitted GLRaV-3 to 2 out of 77 recipient plants and GVA to 1 out of 38, whereas GLRaV-1 was not transmitted. Therefore, this species was not considered a threat to grapevines in northern Italy. Similarly, in our infectivity experiments, the rate of active *H. bohemicus* positive for at least one of the viruses reached 88%, while nymphs from Bennwihr population were able to transmit GLRaV-3 to 12% of recipient vines at most. Comparatively, for the other mealybug species *Ph. aceris* present in our region, the maximal rates of GLRaV-1 and GVA transmission, using only 20 L1 per recipient plant, reached 90% and 100% with a 48 h AAP followed by a 48 h IAP [13].

### 4.5. Acquisition and Inoculation Periods

Our transmission experiments with varying AAP and IAP showed that the *H. bohemicus* L1 nymphs were able to acquire and inoculate GLRaV-3 and GVA after as little as 1 h AAP or 1 h IAP, while at least 6 h AAP and 1 h IAP were necessary for GLRaV-1 transmission. GVA and GLRaV-1 RNAs were already detected in batches of the L2 tested after 1 h AAP. Such short durations are similar to results obtained with other vector species and add further evidence for a semi-persistent mode of transmission of these viruses by mealybugs. For *Pseudococcus longispinus* (Targioni Tozzetti), La Notte et al. [8] found that the shortest AAP and IAP for a successful transmission of GVA were 15 and 30 min, respectively. With the same mealybug species, Krüger et al. [10] obtained transmission events with 15 min AAP and 1 h IAP, and a retention period of 3 days using starving or feeding insects. With *Planococcus ficus* (Signoret) and GLRaV-3, acquisition and inoculation could occur within 1 h [9]. These results correspond to estimates, as nymphs are likely to require some time to reach the phloem after their contact with the leaf. Further work should use the electropenetrography (EPG) method to evaluate more precisely the time required to reach phloem tissues, as previously reported for a few other mealybug species [42,43,44,45,46,47]. The acquisition and inoculation of phloem-restricted viruses are known to occur during the sustained ingestion phase (e.g., [48]). For *Phenacoccus manihoti* Matile-Ferrero, the minimal time to reach the phloem was 1.5 h on a favourable host, and 2.8 h on an unfavourable host [49]. *Ph. aceris* L3 nymphs reached the phloem of infected plants in a mean time of 2.6 h [47]. The shorter minimal AAP obtained by La Notte et al. [8] and Krüger et al. [10] for *Ps. longispinus* could be due to species difference and to the higher number of mealybugs used, which increased the probability of acquisition by the fastest individual in the batch.

### 4.6. Virus Retention

Finally, in our retention experiments, virus detections using RT-PCR showed that the *H. bohemicus* L2 removed from a GLRaV-1 and -3, and GVA infected source grapevines tested positive for viruses until at least 40 days after their transfer onto potatoes, and for 35 days when starved. These results contrast with the few days of retention generally reported in mealybugs [9,10,24,50]. However, detection of viral RNA by RT-PCR neither implies full conservation, thus infectiousness of the virions, nor virus transmission; moreover, it does not inform us as to the virus’s location inside the vector. For instance, *Ph. aceris* lost virus and infectivity for GVA and GLRaV-1 at 5 and 7 days, respectively, after leaving an infected source [47]. *Pl. ficus* nymphs retained GLRaV-3 RNA for at least eight days when feeding on a virus non-host and for two days when starving, and were then capable of transmitting it successfully to healthy grapevines [10]. GLRaV-3 RNA was detected in L1 nymphs of *Pseudococcus calceolariae* (Maskell) up to 16 days on non-*Vitis* plant hosts, but not after 20 days [51]. Moreover, GLRaV-3 was transmitted to grapevines even after the inoculating mealybugs were sustained on white clover plants for 11 days. These different results raise the additional potential effect of host plant on virus retention. Most of our *H. bohemicus* L2 nymphs overwintering on infected grapevines tested positive for viral RNA from December to mid-April. Yet, these nymphs were embedded alone or together inside a powdery wax cocoon under bark layers and could not reach the phloem. Similarly, Le Maguet [52] detected viral RNA continuously in overwintering *Ph. aceris* nymphs from December to February. As in the case of *H. bohemicus*, no virus transmission occurred after infectivity experiments conducted with overwintering *Ph. aceris*, which calls into question their infectiousness despite the detection of viral RNA sequences.

*To conclude*, we provide further findings on the interaction between *H. bohemicus* and grapevine ampelo- and vitiviruses. We observed that its vector ability can vary locally between populations. The duration of virus acquisition and inoculation were found to be only a few hours, in agreement with published evidence of a semi-persistent transmission mode. Mealybugs removed from infected grapevines, then placed on non-host plants or starved, tested positive for viral RNA until 40 and 35 days, respectively, much longer times than those reported for other mealybug species, but this does not imply that they were still infective. Moreover, as with *Ph. aceris*, viral RNA remained detectable in most *H. bohemicus* nymphs overwintering under the trunk bark of infected grapevines, without any feeding opportunity. Further research is needed to characterise the variability in vector efficiency between mealybug populations by clarifying virus–vector interactions and factors affecting their respective virus transmission efficiencies, including genetics.

## Figures and Tables

**Table 1:** Location of the plots sampled for *Heliococcus bohemicus* populations and proportion of viruses among grapevines tested in ELISA for GLRaV-1, -2, and -3, and GVA.

Locality	Latitude	Longitude	Variety	No. Vines	Negative	Positive for			
				Tested		GLRaV-1	GLRaV-2	GLRaV-3	GVA
Bennwihr	48°08′17.7″ N	7°19′06.6″ E	Pinot noir	142	48	33	2	90	6
Colmar	48°06′13.7″ N	7°20′24.6″ E	Riesling	12	10	1		1	
Kientzheim	48°08′24.8″ N	7°16′20.6″ E	Riesling	24	2	22			22
Nothalten	48°21′31.7″ N	7°24′39.7″ E	Riesling	704	282	389	10	110	138
Nothalten	48°21′31.6″ N	7°24′36.0″ E	Pinot noir	275	177	63	1	42	8
Ribeauvillé	48°11′55.6″ N	7°20′11.4″ E	Riesling	21	17	2		2	3
Turckheim	48°05′41.8″ N	7°16′34.2″ E	Sylvaner	14	2	12		1	9

**Table 2:** Numbers of recipient vines used in infectivity experiments, according to their cultivar and the origin of source vines.

Source Vines		Recipient Vines				
Locality	Variety	P114	P115	Pinot Blanc	Pinot Noir	Total
Bennwihr	Pinot noir		104			104
Colmar	Riesling	1	3		4	8
Kaysersberg	Riesling	1			3	4
Turckheim	Sylvaner				2	2
Nothalten	Pinot noir	14	13	3	48	78
Nothalten	Riesling	3	4			7
Ribeauvillé	Riesling	3		1	6	10
Total recipient vines		22	124	4	63	213

**Table 3:** Virus detection by RT-PCR in batches of *Heliococcus* *bohemicus* L2 nymphs and adult males collected on different infected vines in vineyards. Detection rates for each virus according to virus combinations in source vines = number of positive nymph batches/number of batches tested. Positive detections are highlighted in bold.

Growth	N°/Batch	Virus	Source Vine Viruses								
Stages	(Mean ± sd)	Detected	GLRaV-1	GLRaV-3	GLRaV-1, -3	GLRaV-1, -2, -3	GLRaV-1,-2, GVA	GLRaV-1, GVA	GLRaV-1, -3, GVA	Total	%
		GLRaV-1	**1/2**		**4/5**	**3/3**		**2/2**	**1/2**	**11/14**	79%
active L2	1 to 74	GLRaV-2				**2/3**				**2/3**	67%
nymphs	(15 ± 17)	GLRaV-3		**10/12**	**3/5**	**1/3**			**1/2**	**15/22**	68%
		GVA						**2/2**	0/2	**2/4**	50%
		≥1 virus	**1/2**	**10/12**	**5/5**	**3/3**		**2/2**	**2/2**	**23/26**	88%
		GLRaV-1	**18/25**		**28/44**	**6/10**	**3/4**	**4/4**	**14/30**	**73/117**	62%
overwintering	2 to 70	GLRaV-2				**2/10**	**1/4**			**3/14**	21%
L2 nymphs	(11 ± 10)	GLRaV-3		**27/40**	**32/44**	**1/10**			**18/30**	**78/124**	63%
		GVA					**1/4**	**1/4**	**7/30**	**9/38**	24%
		≥1 virus	**18/25**	**27/40**	**41/44**	**7/10**	**3/4**	**4/4**	**23/30**	**123/157**	78%
	1 to 13	GLRaV-1	0/1							0/1	0%
winged males	(6 ± 5)	GLRaV-3		0/3						0/3	0%

**Table 4:** Monthly detection rates by RT-PCR for at least one virus in batches of *Heliococcus* *bohemicus* L2 nymphs during their overwintering under vine bark (number of positive nymph batches/number of batches tested).

Month	December	January	February	March	April
Positive batches/batches tested	23/30	27/37	30/34	25/31	18/25
Proportion	77%	73%	88%	81%	72%

**Table 5:** Transmission rates in infectivity experiments with *Heliococcus* *bohemicus* L1 and L2 nymphs from five locations (Colmar, Kientzheim, Turckheim, Nothalten, and Ribeauvillé), according to virus combinations in source plants. (Number of positive vines/number of inoculated recipient vines).

	No./Plant	Virus	Source Vine Viruses						
GrowthStage	(Mean ± sd)	Transmission	GLRaV-1	GLRaV-3	GLRaV-1, -2, GVA	GLRaV-1, -3	GLRaV-1, GVA	GLRaV-1, -3, GVA	Total
		GLRaV-1	0/6			0/1	0/11	0/3	0/21
L1	2 to 100	GLRaV-3		0/5		0/1		0/3	0/9
	(34 ± 35)	GVA					0/11	0/3	0/14
		Healthy sources							0/20
		GLRaV-1	0/22		0/6	0/9	0/24	0/6	0/67
L2	1 to 60	GLRaV-2			0/6				0/6
	(20 ± 16)	GLRaV-3		0/19		0/9		0/6	0/34
		GVA			0/6		0/24	0/6	0/36
		Healthy sources							0/26

**Table 6:** Transmission rates in infectivity experiments with *Heliococcus* *bohemicus* nymphs from Bennwihr, according to virus combinations in source plants. (Number of positive vines/number of inoculated recipient vines). Virus transmissions are highlighted in bold.

Growth	No./Plant	Virus	Source Vine Viruses					
Stage	(Mean ± sd)	Transmission	GLRaV-1	GLRaV-3	GLRaV-1, -2, -3	GLRaV-1, -3	GLRaV-1, -3, GVA	Total
L1	100	GLRaV-3		**2/2**				**2/2**
		GLRaV-1	0/4		**1/12**	0/38	0/9	**1/63**
active L2	2 to 100	GLRaV-2			0/10			0/10
	(30 ± 21)	GLRaV-3		**3/41**	0/12	**6/38**	**1/9**	**10/100**
		GVA					0/9	0/9
		Healthy sources						0/13
over-		GLRaV-1	0/3		0/3	0/7	0/7	0/20
wintering	9 to 72	GLRaV-2			0/3			0/3
L2	(33 ± 19)	GLRaV-3		0/8	0/3	0/7	0/7	0/25
		GVA					0/7	0/7

**Table 7:** Cross transmission experiments by *Heliococcus* *bohemicus* L2 nymphs according to the origins of mealybug populations and source vines. For each virus, transmission rate = number of positive vines/number of inoculated vines. Virus transmissions are highlighted in bold.

		Source Plants				Transmission Rates		
Date	*H. bohemicus* Origin	Location	Variety	Virus Content	Test Plants	GLRaV-1	GLRaV-3	GVA
2/6/14	Turckheim	Bennwihr	Pinot noir	GLRaV-1, -3, GVA	P115	0/14	0/14	0/14
2/6/14	Ribeauvillé	Bennwihr	Pinot noir	GLRaV-1, -3, GVA	P115	0/11	0/11	0/11
1/10/13	Bennwihr	Nothalten	Riesling	GLRaV-1, -3, GVA	P115	0/9	0/9	0/9
1/10/13	Bennwihr	Nothalten	Pinot noir	GLRaV-1, -3	P115	0/17	0/17	-
26/6/13	Bennwihr	Nothalten	Pinot noir	GLRaV-1, -3	Pinot noir	0/6	**2/6**	-
26/6/13	Bennwihr	Nothalten	Pinot noir	GLRaV-3	P115	-	**2/5**	-
1/10/13	Bennwihr	Nothalten	Pinot noir	GLRaV-3	P115	-	0/15	-

**Table 8:** Detection rates of GLRaV-1 and -3, and GVA in L1 nymphs of *Heliococcus bohemicus*, after AAP from 24 h to 72 h, with 5, 10, or 20 nymphs per batch. Number of positive batches/number of batches tested. Positive detections are highlighted in bold.

	AAP		24 h			48 h			72 h	
	L1 Numbers	5 L1	10 L1	20 L1	5 L1	10 L1	20 L1	5 L1	10 L1	20 L1
Virus	GLRaV-1	**2/5**	**4/5**	**3/4**	**3/5**	**5/5**	**5/5**	**4/5**	**5/5**	**5/5**
detection	GLRaV-3	0/5	**2/5**	**2/4**	0/5	**3/5**	**4/5**	0/5	**1/5**	**2/5**
rates	GVA	**1/5**	**2/5**	**3/4**	**3/5**	**5/5**	**5/5**	**1/5**	**3/5**	**5/5**

**Table 9:** Virus transmission rates of *Heliococcus bohemicus* L1 nymphs, according to AAP/IAP combinations. AAP on accession Y258. For each virus, transmission rate = number of positive vines/number of inoculated vines. Transmission events are highlighted in bold.

Time		Transmission		Rate
AAP	IAP	GLRaV-1	GLRaV-3	GVA
1 h	1 h	0/5	0/5	0/5
1 h	6 h	0/5	0/5	0/5
1 h	24 h	0/5	**1/5**	**1/5**
6 h	1 h	**2/5**	0/5	0/5
6 h	6 h	0/5	0/5	0/5
6 h	24 h	0/5	0/5	0/5
24 h	1 h	0/5	**1/5**	**1/5**
24 h	6 h	**1/5**	**1/5**	**1/5**
24 h	24 h	0/5	**1/5**	**1/5**

**Table 10:** Detection rates of GLRaV-1 and -3, and GVA in *Heliococcus bohemicus* L2 batches, after 3 days acquisition on accession Y258 (infected by GLRaV-1 and -3, and GVA), and various times starving or confined on potato (number of virus positive batches/number of surviving batches). Positive detections are highlighted in bold.

	Diet					
	Starving			On Potato		
	Virus Detection Rate			Virus Detection Rate		
Retention days	GLRaV-1	GLRaV-3	GVA	GLRaV-1	GLRaV-3	GVA
7	**5/5**	**4/5**	**4/5**	**4/5**	**1/5**	**2/5**
8	**5/5**	**3/5**	**4/5**	**5/5**	**5/5**	**2/5**
9	**3/5**	**4/5**	**1/5**	**4/5**	**4/5**	**4/5**
10	**4/5**	**4/5**	**3/5**	**5/5**	**5/5**	**4/5**
11	**3/5**	**2/5**	**1/5**	**3/5**	**2/5**	**1/5**
12	**5/5**	**2/5**	**3/5**	**5/5**	**5/5**	**4/5**
13	**4/5**	**5/5**	**5/5**	**5/5**	**4/5**	**3/5**
14	**5/5**	**3/5**	**4/5**	**3/5**	**3/5**	**2/5**
15	**3/5**	**1/5**	**1/5**	**3/5**	**1/5**	**1/5**
20	**4/4**	**3/4**	**4/4**	**5/5**	**5/5**	**3/5**
25	0/5	0/5	0/5	**1/5**	**1/5**	0/5
30	**5/5**	**5/5**	**4/5**	0/5	**1/5**	0/5
35	**5/5**	**5/5**	**3/5**	**-**	**-**	**-**
40	-	-	-	**5/5**	**5/5**	**3/5**
50	-	-	-	0/4	0/4	0/4
60	-	-	-	0/5	0/5	0/5

## Data Availability

Data supporting the results reported are available on request.
